# Robust fabrication of thin film polyamide-TiO_2_ nanocomposite membranes with enhanced thermal stability and anti-biofouling propensity

**DOI:** 10.1038/s41598-017-18724-w

**Published:** 2018-01-15

**Authors:** Behnam Khorshidi, Ishita Biswas, Tanushree Ghosh, Thomas Thundat, Mohtada Sadrzadeh

**Affiliations:** 1 Department of Mechanical Engineering, 10-367 Donadeo Innovation Center for Engineering, Advanced Water Research Lab (AWRL), University of Alberta, Edmonton, T6G 1H9 AB Canada; 2 Department of Mechanical Engineering, Biomicrofluidics Lab, University of Alberta, Edmonton, AB T6G 1H9 Canada; 3 Department of Chemical & Materials Engineering, 13-287 Donadeo Innovation Centre for Engineering, University of Alberta, Edmonton, T6G 1H9 AB Canada

## Abstract

The development of nano-enabled composite materials has led to a paradigm shift in the manufacture of high-performance nanocomposite membranes with enhanced permeation, thermo-mechanical, and antibacterial properties. The major challenges to the successful incorporation of nanoparticles (NPs) to polymer films are the severe aggregation of the NPs and the weak compatibility of NPs with polymers. These two phenomena lead to the formation of non-selective voids at the interface of the polymer and NPs, which adversely affect the separation performance of the membrane. To overcome these challenges, we have developed a new method for the fabrication of robust TFN reverse osmosis membranes. This approach relies on the simultaneous synthesis and surface functionalization of TiO_2_ NPs in an organic solvent (heptane) via biphasic solvothermal reaction. The resulting stable suspension of the TiO_2_ NPs in heptane was then utilized in the interfacial (*in-situ*) polymerization reaction where the NPs were entrapped within the matrix of the polyamide (PA) membrane. TiO_2_ NPs of 10 nm were effectively incorporated into the thin PA layer and improved the thermal stability and anti-biofouling properties of the resulting TFN membranes. These features make our synthesized membranes potential candidates for applications where the treatment of high-temperature streams containing biomaterials is desirable.

## Introduction

Due to drastic world population growth, rapid industrialization and widespread climate change in recent years, the world is facing the threat of a water scarcity. Today, more than 1.8 billion people, around one-fifth of the world’s population, live in areas with severe water shortage; where poor access to clean and safe drinking water causes several million deaths every year^1–4^. Hence, the fulfilment of the critical global water demand with the energy-efficient and cost-effective technologies to produce and recycle high-quality water has become a high priority^5,6^.

Membrane technology is being widely used in many water treatment processes including sea and brackish water desalination as well as the industrial and municipal wastewater reclamations^7,8^. Currently, the majority of commercial desalination and water treatment plants use thin film composite (TFC) nanofiltration (NF) and reverse osmosis (RO) membranes^9–11^. A TFC membrane consists of a thin selective layer, usually made of polyamide (PA), over a microporous support layer (e.g., polyethersulfone (PES), backed by a polyester fabric mesh)^12,13^. The PA layer is prepared by interfacial polymerization (IP) reaction between a polyfunctional amine, e.g., m-phenylenediamine (MPD), and a polyfunctional acyl chloride molecule, such as trimesyol chloride (TMC)^14,15^. The multilayer structure of the TFC membranes allows the unique opportunity to tune and enhance the properties of the selective and supportive layers independently with the use of novel materials and advanced synthesis methods^16,17^. Regarding that, considerable effort has been devoted to integrate the advances in nanotechnology with the classical synthesis procedure of the polymeric membranes with the aim of fabricating novel multifunctional nanocomposite membranes^18,19^. The first instance of such work was reported for gas separation by adding zeolite nanofillers into silicone rubber in 1973^20^. Later, in 2005, the application was extended to water filtration with the incorporation of zeolite-A NPs into a thin-film nanocomposite (TFN) PA membrane^21^. Since then, a variety of nanoparticles (NPs), including organic (carbon-based nanomaterials such as carbon nanotube, graphene, and graphene oxide) and inorganic (zeolite, silica, metal and metal oxide) nanofillers, have been utilized to fabricate TFN membranes for diverse applications of gas separation, pervaporation, and water purification processes^22–27^.

Recently, titanium dioxide (TiO_2_) has attracted considerable attention for improving the permselectivity and antifouling propensity of the TFN PA membranes owing to its low production cost, high chemical and thermal stability and, most importantly, its photocatalytic activity upon UV irradiation^28–31^. There are two main approaches that have been widely utilized to incorporate TiO_2_ NPs in TFN PA membranes: (i) attachment via self-assembly to the PA surface^32–34^ and (ii) *in-situ* integration into PA matrix during the IP reaction^35–37^. The former involves dip coating of a prepared TFC membrane into a TiO_2_ NP suspension. In the second method the TiO_2_ NPs are directly dispersed in one of the reacting monomer (either MPD-aqueous or TMC-organic) solutions prior to IP reaction^38^. The self-assembly is highly efficient in modifying the surface properties of the membrane with easy implementation since the NPs are directly deposited on the membrane surface^39^. However, the weak attachment between the TiO_2_ NPs and the host PA membrane surface may cause the leaching of the NPs during the filtration process and thus limits its application for a long-term operation^40^. The significant advantage of the *in-situ* integration method is to resolve this problem by the entrapment of the TiO_2_ NPs within the PA matrix during the IP reaction^41^. However, the critical challenge with this method is that the fabrication of a defect-free PA film requires first, synthesis of nanosized TiO_2_ NPs and second, preparation of a stable dispersion of the TiO_2_ NPs in the monomer solutions^42^.

In recent years, various approaches to effectively incorporate NPs in the thin PA layer via the *in-situ* polymerization have been reported^43,44^. Due to the strong interaction of most nanomaterials with polar solvents, much of the research up to now has been restricted to the dispersion of NPs in MPD-aqueous solution^45^. A possible weakness of this approach, however, is that NPs cannot be integrated into the topmost PA layer and, as a result, their properties are not present in the membrane/water interface^46^. Given that, it is preferable to have NPs dispersed in TMC-organic solvent (e.g. heptane). In such cases, however, the aggregation of TiO_2_ NPs becomes more challenging as the thermodynamics of the resulting multiphase system are inherently unstable.

In the present work, we report a highly robust and efficient method for incorporation of TiO_2_ NPs into the PA matrix. The synthesis and surface modification of the NPs were carried out simultaneously using oleic acid (OA) in heptane via a biphasic solvothermal (BST) reaction. The resulting stable TiO_2_ suspension in heptane was then added to TMC-heptane solution to fabricate a TFN PA membrane. It is anticipated that the stable dispersion of nanosized TiO_2_ particles with a very low aggregation rate in the TMC-heptane solution can efficiently result in nanocomposite PA films with enhanced thermomechanical, antibacterial, and permeation performance.

## Results and Discussion

**Figure 1:**
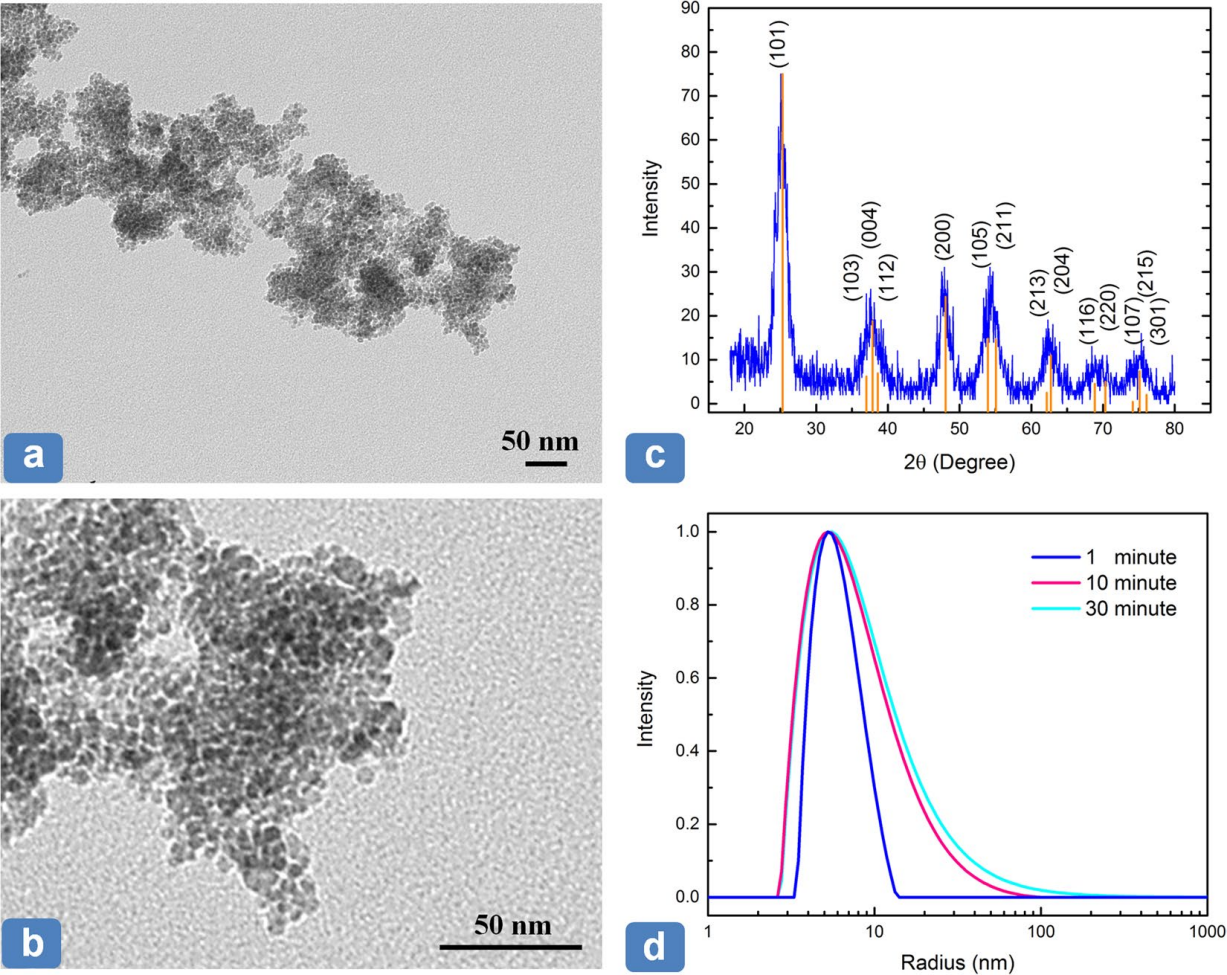
(**a**) and (**b**) TEM images of the synthesized TiO_2_ NPs presenting the size of dried nanoparticles, (**c**) XRD spectrum of TiO_2_ NPs showing their anatase crystalline structure, (**d**) DLS measurement of TiO_2_ NPs capped with OA presenting their stability and size distribution in heptane.

TiO_2_ NPs, synthesized using the BST reaction, were characterized applying transmission electron microscopy (TEM), X-ray photoelectron spectroscopy (XRD) and dynamic light scattering (DLS) techniques. The TEM images in panels (a) and (b) of Fig. 1 illustrate that the TiO_2_ NPs have a nano size core in the range of 5 to 10 nm. Regarding crystalline shape, TiO_2_ NPs are generally available in three structures, namely anatase, rutile, and brookite. The prepared TiO_2_ NPs mainly have the anatase structure as evidenced by the characteristic peaks at 2θ degree of 25.3°, 37.8°, and 48.1° in the XRD spectra (panel c). Theses peaks correspond to the (101), (004) and (200) planes, respectively^28^. The anatase structure is known to provide higher photocatalytic activities, as will be discussed later. The size distribution and aggregation rate of dispersed TiO_2_ NPs in heptane were evaluated by DLS technique. The size measurement was conducted three times over a period of 30 minutes. Panel (d) in Fig. 1 demonstrates that the TiO_2_ NPs were highly stable in heptane with the average size of less than 10 nm, starting from 7.1 nm (after 1 minute) and reaching to 8.5 nm after 30 minutes. The high stability of the TiO_2_ NPs in heptane is essential for the synthesis of defect-free PA films.

**Figure 2:**
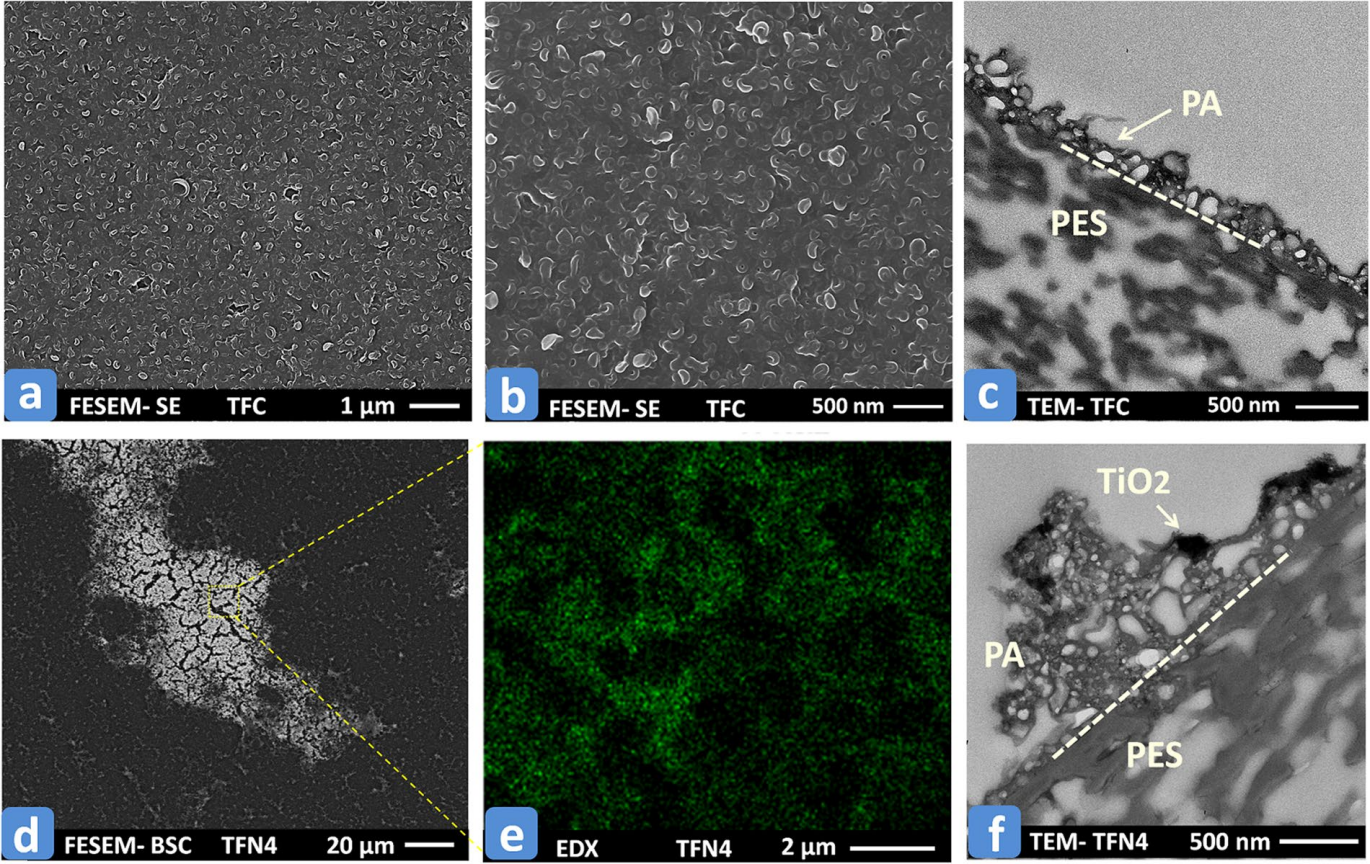
(**a**) and (**b**) FESEM images of the base TFC membrane; (**c**) TEM images of the TFC membrane; (**d**) FESEM images with BSE detector of the TFN4 membrane showing the TiO_2_-rich spots brighter than the other regions; (**e**) EDX color map of Ti element at the surface of TFN4 membrane; (**f**) TEM images of the TFN4 membrane.

Figure 2 illustrates the surface and cross-sectional images of the base TFC and TFN4 membranes (See Table 1 in the Materials and Methods section for the synthesis conditions of TFC and TFN membranes). The surface images were obtained via field emission scanning electron microscopy (FESEM). The FESEM and TEM images in panels (a), (b), and (c) demonstrate that the PA layer of the base TFC membrane possesses a ridge-and-valleys morphology with multiple internal nano- and microvoids^47,48^. In contrast to the homogeneous surface feature of the base TFC membrane, the FESEM image of TFN4 membrane obtained using back-scattered electron detector (BSE) in panel (d) reveals the formation of multiple large islands at the membrane surface. These islands were blend of PA with a high concentration of TiO_2_ NPs. The elemental color map in panel (e), which was obtained by energy-dispersive X-ray spectroscopy (EDX), also confirmed a good distribution of TiO_2_ NPs within the PA structure. The TEM cross-sectional images of the TFN4 membrane (panel f) show that the TiO_2_ NPs were mainly integrated into the topmost layer of the PA film. It is highly desirable as it enables tuning of the surface and bulk properties of the nanocomposite membranes. The chemical properties of the synthesized membranes were also evaluated using attenuated total reflection-Fourier transform infrared (ATR-FTIR) spectroscopy and EDX techniques and the results are presented in Figures S1 and S2 in the Supporting Information. The ATR-FTIR spectra demonstrated the presence of PA chemical functional groups that include C=O stretching vibration (amide I bands) and the C-N stretching vibration (amide II bands). The EDX spectroscopy showed a distinct peak for titanium at the membrane surface that confirmed effective addition of the TiO_2_ NPs to the PA layer during the IP reaction. The thermomechanical stability of the base TFC and TFN4 membranes was also tested by thermogravimetric analysis (TGA) and the results are presented in Figure S3 in the Supplementary Information. Based on this figure, the onset of intense degradation temperature of the composite membrane has slightly increased from 530 °C for the TFC to 550 °C for TFN4. The improved thermal stability is attributed to the decreased polymer chain mobility due to the presence of nano-sized and homogeneously distributed TiO_2_ NPs^49^.

**Table 1:** Concertation of MPD, TMC and TiO2 NPs for the fabrication of TFC and TFN membranes. The invariant synthesis conditions were: 0.2 wt.% SDS, 1 wt.% CSA, 1 wt.% TEA in MPD-water solution, 30 sec IP reaction, 4 minutes heat curing at 60 °C.

Membrane	MPD concentration in DI water (wt.%)	TMC concentration in heptane (wt.%)	Volume of TiO_2_ NP suspension added to TMC-heptane solution (µl)	Estimated concentration of TiO_2_ NPs in TMC-heptane solution (wt.%)
TFC	2	0.2	0	0
TFN1	2	0.2	125	0.006
TFN2	2	0.2	250	0.0124
TFN3	2	0.2	375	0.0185
TFN4	2	0.2	500	0.0245

The permeation performance of a TFC membrane highly depends on its physicochemical and structural characteristics such as the thickness and crosslinking density of the PA layer, the complex interior free volumes, the surface charge, and hydrophilicity of the membrane^50–53^. In the case of TFN membranes, the surface and bulk properties of the incorporated nanomaterials as well as their interaction with the host polymer matrix have also a significant influence on the final transport properties of the TFN membranes.

**Figure 3:**
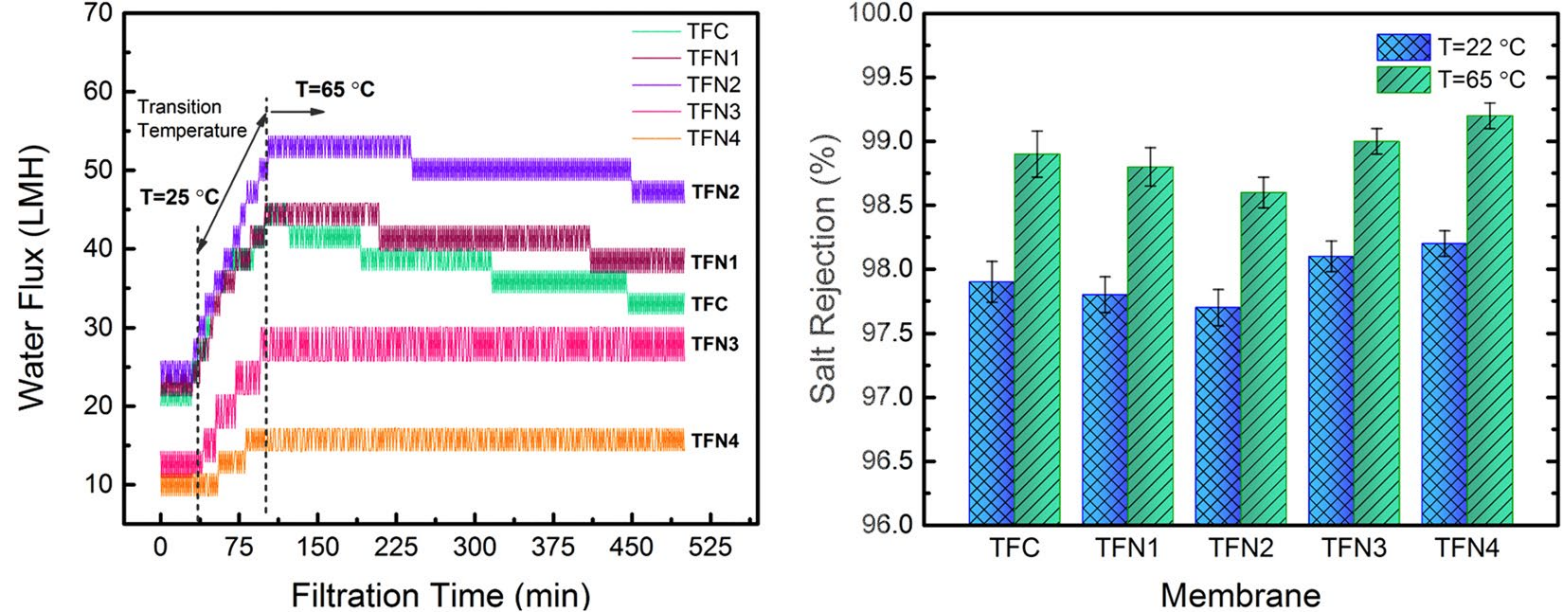
Water permeation and salt rejection of the synthesized TFC and TFN membranes at 25 °C and 65 °C showing the effect of TiO_2_ NPs on permselectivity and thermal stability of the TFN membranes. Operating conditions: 220 ± 5.0 psi of transmembrane pressure and 1.0 ± 0.1 LPM of feed flow rate.

The water flux and salt rejection of the synthesized TFC and TFN membranes are presented in Fig. 3. To evaluate the thermal stability of the membranes, ffiltration tests were initially conducted at room temperature (25 °C) and then elevated to 65 °C. The comparison between the water permeation results at room temperature revealed that the addition of TiO_2_ NPs into the PA layer initially improved the water flux of the resulting TFN membrane (22.7 LMH and 24.3 LMH for TFN1 and TFN2, respectively, compared to 21.5 LMH for base TFC membrane). Further incorporation of TiO_2_ NPs, however, resulted in TFN membranes with lower water flux (10.7 LMH and 10.0 LMH for TFN3 and TFN4 membranes, respectively). The higher water flux of the TFN1 and TFN2 membranes than that of TFC membrane suggests the existence of an optimum loading of the TiO_2_ NPs for the fabrication of high throughput TFN membranes. Regarding salt rejection, all the synthesized membranes provided greater selectivity than 97% of NaCl in water. The salt rejection of the base TFC membrane and TFN1 and TFN2 membranes were almost similar; however, TFN3 and TFN4 membranes provided a higher salt rejection following the typical trade-off relation between the water flux and salt rejection. The lower water flux and higher salt rejection of TFN3 and TFN4 can be attributed to the reduced internal free volumes within the PA matrix by the integration of well-dispersed nanosize TiO_2_ particles. The presence of these NPs may have restricted the pathways for the transport of water and solute molecules through the TFN membranes.

By increasing the temperature of the feed solution (2000 ppm NaCl), all the synthesized membranes provided more water flux with a maximum value at 65 °C. The progressive increase in the permeation rate of the membranes with temperature can be attributed to the swelling of the PA layer as well as the higher diffusion coefficient of water molecules through the swelled membrane^54–57^. When the filtration test continued for a longer time at 65 °C, the base TFC membrane showed a stepwise flux decline from 43.5 LMH to 33 LMH over 6 hours of filtration test. The observed flux decline became milder for the case of the TFN1 and TFN2 and was completely overcome in the case of TFN3 and TFN4 in which higher concentration of TiO_2_ NPs was integrated. A possible explanation for this stepwise flux decline may be the compaction of the swelled PA film under high temperature and pressure (65 °C and 220 psi, respectively). Hence, it can be concluded that the addition of the TiO_2_ NPs could effectively improve the thermomechanical stability of the TFN membranes by limiting the chain mobility of the PA matrix at high temperatures. Regarding the salt rejection, all the synthesized membranes showed higher rejection percentage at 65 °C as compared to room temperature, particularly for the case of TFN3 and TFN4 where the salt rejection was as high as 99.2% and 99.4%, respectively. The high salt rejection and stable water permeation of TFN3 and TFN4 are highly desirable for the applications that robust membrane performance and high-quality water is required under high temperatures of feed streams. Such an application can be found in Steam-Assisted Gravity Drainage (SAGD) process of oil recovery where the temperature of the produced water is as high as ∼150 °C. In order to reuse and recycle the produced water, a significant amount of energy is wasted by cooling down the produced water to ∼60 °C in water treatment processes, follwed by re-heating to ∼200 °C in steam generators^58,59^. Thermally tolerant membranes have the potential to integrate into water treatment facilities and reduce the boiler heating requirements and greenhouse gas production in oil recovery plants.

**Figure 4:**
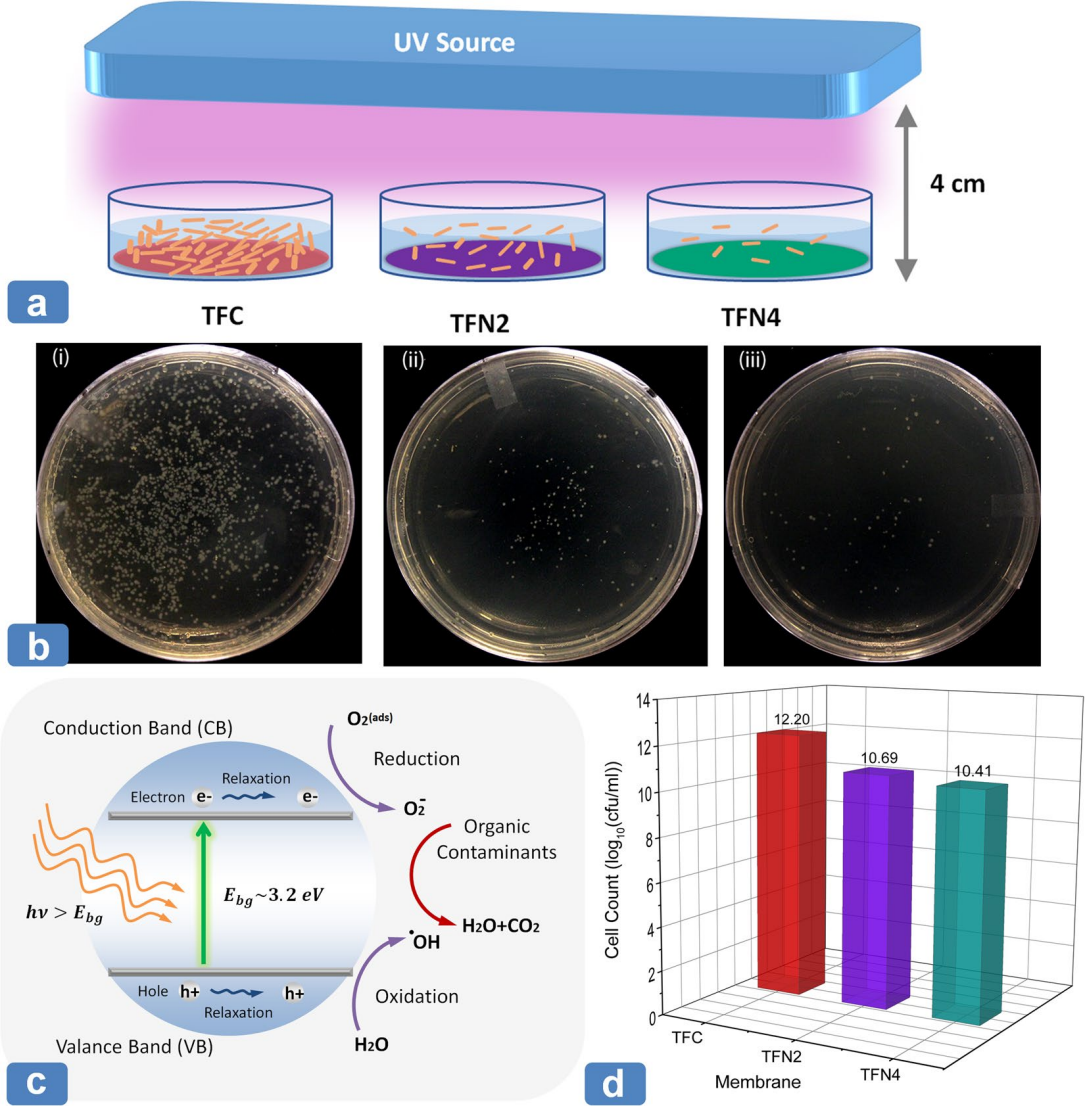
(**a**) Schematic view of the measurement of the antibacterial activity of TFN membranes; (**b**) Images of the *E. coli* colonies formed in the plate of UV-treated (i) TFC, (ii) TFN2 and (iii) TFN4 membranes; (**c**) Mechanism for photocatalytic activity of TiO_2_ NPs under UV irradiation; (**d**) Number of *E. coli* colonies counted on the plate of TFC, TFN2 and TFN4 membranes after 30 minutes of UV irradiation.

The photocatalytic activity of TiO_2_ has made it a potential material to be used in a wide range of applications including food and medical industry, solar energy conversion and water purification. Under the UV light (Fig. 4a), the TiO_2_ NPs generates active radical species (such as hydroxyl and superoxide radicals) which decompose organic matter and inactivates living organism like bacteria in the water (Fig. 4c)^60,61^. A moderate band gap (~3.2 ev) along with low toxicity has made TiO_2_ NPs promising nanofillers for water treatment applications, particularly for mitigating the bio- and organic fouling^62–64^.

The antibacterial activity of the synthesized TFN membranes in this study was evaluated by counting the number of bacteria (*E. coli*) colonies formed over the UV-illuminated membranes^65,66^. Figure 4b illustrates the image of three plates of base TFC, TFN2 and TFN4 membranes after 30 minutes of UV irradiation. As can be observed, the exposure of the UV light on TFN membranes effectively lowered the viability of the *E. coli* bacteria. The number of bacteria colonies dramatically decreased from 12.2 $\,{\mathrm{log}}_{10}({\rm{cfu}}/{\rm{ml}})$ for the base TFC membrane to 10.7 $\,{\mathrm{log}}_{10}({\rm{cfu}}/{\rm{ml}})$ for the TFN2 membrane as presented in Fig. 4d. Further increase in the concentration of TiO_2_ NPs in TFN4 membranes provided a slightly higher antibacterial efficiency compared to TFN2 membrane. The distinct difference between the numbers of bacteria colonies on the surface of the TFN membranes compared to the base TFC membrane shows the high photocatalytic activity and thus the antibacterial efficiency of the integrated TiO_2_ NPs within the PA network. This property is highly beneficial for those membrane-based separation processes where mitigation of biofouling and organic deposition at the membrane surface is essential.

## Discussion

The permeation properties and the fouling propensity of TFC and TFN membranes are strongly influenced by the surface physicochemical characteristics (such as morphology, thickness, surface roughness, charge density, the degree of cross-linking, and hydrophilicity) as well as the complex inter-connected free volumes within the PA structure. In the present study, the main objective was to incorporate the TiO_2_ NPs into the PA matrix to improve the thermal stability and antibiofouling properties of the resulting TFN membranes. The permeation results at the elevated temperature (Fig. 3) revealed that the thermal stability of all TFN membranes improved as compared to the base TFC membrane. The higher thermal stability is likely due to formation of a denser structure by the addition of nanosized TiO_2_ NPs into the PA structure. This result can be confirmed by comparing the water permeability of the TFN4 and TFC membranes where the addition of high concentration of TiO_2_ NPs led to a denser membrane with lower water flux but higher salt rejection and greater thermal stability. However, there was an optimal concentration of NPs in which the synthesized TFN2 membrane showed both higher water flux and thermal stability than the base TFC membrane. Furthermore, the antibiofouling test with *E. coli* bacteria under the UV irradiation revealed that all the TFN membranes demonstrated higher photocatalytic activity and antifouling propensity compared to the base TFC membrane.

The main two challenges of using biphasic solvothermal method for the synthesis of TiO_2_ NPs are related to the high energy requirement and scaling up the mass production of NPs. Solvothermal synthesis procedure is typically more energy-intensive than the conventional hydrolysis (such as sol-gel) methods due to the high-temperature treatment of solvents (often higher than 140 °C) for a long period. However, it must be noted that the NPs synthesized by the conventional hydrolysis methods also require thermal post-treatment to obtain proper crystalline shape. The production rate of NPs in the hydrolysis process is also higher than the solvothermal method; however, the size of the NPs, which is highly critical in the present study, is more controllable in the solvothermal method. It is worth mentioning that, one-pot synthesis procedure without the need for the post-synthetic crystallization annealing provides a great potential for scaling up the hydrothermal and solvothermal methods^67–69^. Finally, in the present study, to make robust TFN membranes, the synthesized NPs were required to be well dispersed in an organic solvent (heptane). Therefore, employing a hydrolysis method requires an additional surface functionalization to make the NPs dispersible in the organic solvent. In order to skip this extra surface functionalization step, the biphasic solvothermal method was applied for *in-situ* synthesis and surface functionalization of the TiO_2_ NPs. The resulting NPs were stable in heptane and were directly added into the TMC-heptane solution for the IP reaction.

## Materials and Methods

### Materials and chemicals

m-phenylenediamine (MPD, ≥99%) and 1,3,5-benzenetricarbonyl trichloride (TMC, 98%) were purchased from Sigma-Aldrich and used as reacting monomers for the synthesis of the PA films. Sodium dodecyl sulfate (SDS), camphorsulfonic acid (CSA), and triethylamine (TEA) were obtained from Fisher Scientific and utilized as the chemical additives in MPD-aqueous solution^70^. Titanium (IV) isopropoxide (98%, density of 0.96 g/ml and molar mass of 284.215 g/mol), oleic acid (OA, ≥99%) and heptane (≥99%) were purchased from Sigma-Aldrich and employed for the synthesis of the TiO_2_ NPs. All chemicals were used as obtained without further purification. Polyethersulfone (PES) microfiltration sheets with average pore size of 100 nm were obtained from Sterlitech Co. (WA, USA) and applied as a supporting layer for the fabrication of the composite membranes.

### Synthesis of TiO_2_ NPs using biphasic solvothermal (BST) reaction

TiO_2_ NPs were prepared using a biphasic solvothermal (BST) reaction where the reaction takes place at the interface of water and organic solutions at an elevated temperature^71,72^. First, a water solution consisting of 20 ml of DI water and 200 µl of TEA (as a pH regulator) was added into a PTFE Teflon liner. Afterward, the organic solution was prepared by adding 800 µl of titanium (IV) isopropoxide and 1200 µl OA to 20 ml of heptane. The heptane solution was sonicated for 30 minutes in an ultrasonic bath (FS30H, Fisher Scientific) and then was poured gently over the water phase in the PTFE liner. The Teflon vessel was placed into a stainless steel autoclave, then sealed and heated in a vacuum oven (Thermo Scientific Heratherm™, USA) for 8 hours at 200 °C. Titanium (IV) isopropoxide reacted with water at the water/heptane interface to generate TiO_2_ as follows^73^:

1 $${\rm{Ti}}{[{\rm{OCH}}{({{\rm{CH}}}_{3})}_{2}]}_{4}+2{{\rm{H}}}_{2}{\rm{O}}\to {{\rm{TiO}}}_{2}+4{({{\rm{CH}}}_{3})}_{2}{\rm{CHOH}}$$

**Figure 5:**
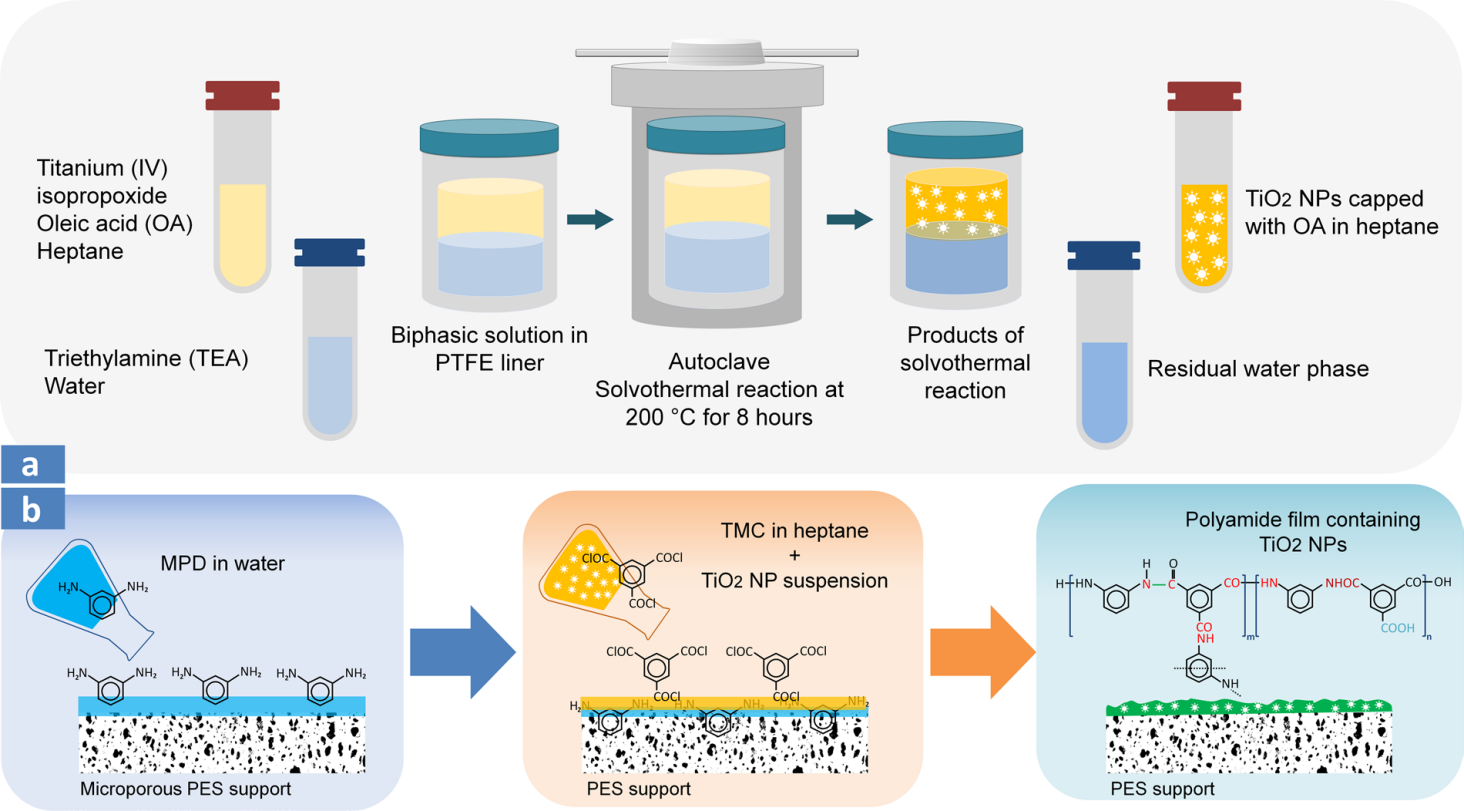
(**a**) Schematic synthesis route of TiO_2_ NPs via biphasic solvothermal (BST) reaction and (**b**) fabrication of TFN membranes via adding TiO_2_ NPs during interfacial polymerization (IP) reaction.

The autoclave was cooled down for 12 hours at room temperature and the supernatant organic solution which contained TiO_2_ NPs capped with OA was carefully extracted. Assuming 100% conversion for the progress of the above reaction, the produced TiO_2_ was found to be 0.01 mg/μl. Figure 5a schematically illustrates the synthesis route of the TiO_2_ NPs using BST reaction.

### Synthesis of TFC and TFN membranes

TFC membranes were prepared by utilizing the interfacial polymerization reaction between MPD-water and TMC-heptane solutions at the surface of the PES microporous layer (Fig. 5b). Initially, the PES substrate was placed between a plexiglass frame and a rubber gasket (15 × 20 cm^2^), which were firmly attached using stainless steel binder clips. Next, the MPD solution containing 2 wt.% MPD, 0.2 wt.% SDS, 1 wt.% CSA, and 1 wt.% TEA was poured on the PES surface and allowed to saturate the PES sheet for 10 minutes. The amine solution was then drained off from the surface, the plexiglass and rubber gasket were disassembled, and the excess MPD droplets were removed from PES surface using a rubber roller. Afterward, the MPD-impregnated PES sheet was again fixed between the plexiglass frame and gasket and 0.2 wt.% TMC solution in heptane was gently poured on the surface. The polymerization reaction was allowed to proceed for 30 seconds, and then the resulting composite PA membrane was thermally cured in a digital oven at 60 °C for 4 minutes^74^. Finally, the membranes were thoroughly washed with DI water and stored in a covered polyethylene tub filled with DI water at 25 °C temperature. To prepare TFN membranes, different volumes of the extracted TiO_2_ NP suspension (125 µl, 250 µl, 325 µl and 500 µl) were added into 20 g of TMC-heptane solution. Table 1 presents the synthesis conditions for fabrication of TFC and TFN membranes.

### Characterization of TiO_2_ NPs and TFN membranes

The crystalline structure of the TiO_2_ NPs was examined by XRD technique (Rigaku XRD Ultima IV, Cu-Kα source, 40 kV, 44 mA). The collected spectrum was analyzed using JADE software. Particle size and stability of the dispersed TiO_2_ NPs in heptane were measured by DLS technique (ALV/CGS-3 compact goniometer, ALV-GmbH, Langen, Germany). Particle size distribution (PSD) of TiO_2_ NPs was extracted by CONTIN analysis through scattering results obtained from He-Ne laser at 632.8 nm. The size of TiO_2_ NPs was also measured using TEM (Philips/FEI Morgagni 268, Netherlands) device. The sample was prepared by placing a drop of TiO_2_-heptane solution on a copper grid. Within a short period, the heptane evaporated and TiO_2_ NPs attached to the supporting copper grid. TEM was also used to obtain the cross-sectional images of the synthesized membranes. Membrane samples were first stained with lead citrate and uranyl acetate, and then embedded in Spurr’s resin. Ultrathin sections of the samples were then prepared using an ultramicrotome (Reichert-Jung Ultracut E, USA) and finally, examined by TEM. The surface morphology of the TFC and TFN membranes was characterized by FESEM (Zeiss Sigma 300 VP) technique. The microscope was also equipped with EDX (Bruker) detector for elemental mapping and phase identification. The samples were carbon-coated and examined with a SE and BSE detectors. The chemical composition of the prepared TFC and TFN membranes were analyzed using ATR-FTIR spectroscopy (DIGILAB FTS 7000 Series, Marlborough, MA). The thermal stability of the composite polymers was evaluated by thermogravimetric analysis (TGA) using TGA-Q500 (TA instrument, USA). The water flux and salt (NaCl) rejection of the prepared membranes were measured using a cross-flow filtration setup (Sterlitech Co., USA). The operating test condition was set to 220 ± 5 psi (1.52 ± 0.03 MPa) of transmembrane pressure and a constant feed flow rate of 1 ± 0.1 Lmin^−1^ (16.7 ± 1.7 cm^3^/s). The filtration test was initially started with feed solution (2000 ppm NaCl) at room temperature for 30 minutes, and then the feed temperature was elevated to 65 °C using a circulating water bath (Isotemp3013, Fisher Scientific). Afterward, the filtration test was continued for 5 hours at 65 °C to evaluate the permeation performance and thermal stability of the membrane at high temperature. The permeate volumetric flux (J_W_) was obtained by measuring the mass of permeate water (Δ*M*) over the specific time (Δ*t*) of data recording (every minute in this work using LabVIEW software) divided by the effective membrane surface area (A = 42 × 10^−4^ m^2^):

2 $${{\rm{J}}}_{{\rm{W}}}=\frac{{\rm{\Delta }}{\rm{M}}}{{\rm{\rho }}.{\rm{A}}.{\rm{\Delta }}{\rm{t}}}$$

where $\rho$ is the density of the permeate water. The apparent salt rejection (*R*) was evaluated using:

3 $${\rm{R}}=(1-{C}_{{\rm{p}}}/{C}_{{\rm{f}}})\times 100$$

where *C*_p_ and *C*_f_ are the salt concentration in the permeate and feed water (2000 NaCl solution), respectively. The values of *C*_p_ and *C*_f_ are obtained using a calibration curve of the solution electrical conductivity measured by a conductivity meter (Accumet AR50, Fisher Scientific).

The antibacterial activity of the membranes was evaluated using Escherichia coli (*E. coli* DH5α) bacteria strain. Bacterial culture was prepared in Luria–Bertani (LB) broth media (Fisher Scientific, Canada) and grown for 36 h at 30 °C in a shake-flask at 180 rpm. A piece of base TFC, TFN2 and TFN4 membranes were cut into 7.5 cm^2^ and placed at the bottom of three separate petri plates (30 mm diaHandheld Lamp, CA, USA) was placedmeter, Fisher Scientific, Canada). Bacteria solution (2 ml each) was poured in the petri plates on top of the membrane surface. UV light lamp (254 nm, UVGL – 58 Handheld Lamp, CA, USA) was placed at a height of 4 cm from the open surface of petri plates (Fig. 4a). The irradiance of the UV light (6 W) was measured as 2.10 mW/cm^2^ (100 C power meter, AB-M Inc. CA, USA). After 30 minute of UV exposer, a 10:1 serial dilution was carried out for each petri plates with LB broth. Then, 250 µl of diluted bacteria solution from each plate was poured in LB agar plate. After overnight incubation at 30 °C, the antibacterial property of the membrane was observed by counting the number of colony formation on the plates.

## Electronic supplementary material

Supplementary information
