# Supplementary material for: Robust fabrication of thin film polyamide-TiO2 nanocomposite membranes with enhanced thermal stability and anti-biofouling propensity
Source: Sci Rep. 2018 Jan 15;8:784. doi: 10.1038/s41598-017-18724-w (PMC5768769; doi:10.1038/s41598-017-18724-w)
Supplement: Supplementary file 1 — Supplementary information [file 41598_2017_18724_MOESM1_ESM.doc]

**Supplementary Information**

**Robust fabrication of thin film polyamide-TiO_2_ nanocomposite membranes with enhanced thermal stability and anti-biofouling propensity**

Behnam Khorshidi^1^, Ishita Biswas^1^, Tanushree Ghosh^2^, Thomas Thundat^2^, Mohtada Sadrzadeh1[[1]](#footnote-2)*

^1^ Department of Mechanical Engineering, 10-367 Donadeo Innovation Center for Engineering, Advanced Water Research Lab (AWRL), University of Alberta, Edmonton, AB, Canada, T6G 1H9

^2^ Department of Chemical & Materials Engineering, 13-287 Donadeo Innovation Centre for Engineering, University of Alberta, Edmonton, AB, Canada, T6G 1H9

The supplementary materials presented here provide more information and details to the chemical characterization of the synthesized TFN and TFC PA membranes. This section includes the ATR-FTIR as well as the EDX analysis of the prepared membranes.

.

The chemical properties of the synthesized membranes were evaluated using Attenuated total reflection-Fourier transform infrared (ATR-FTIR) spectroscopy and EDX techniques. Figure S1 presented the ATR-FTIR spectra of the TFC and TFN membranes. These spectra demonstrate the chemical functional groups present in both top PA skin layer and the bottom PES substrate due to the high penetration depth of the IR beam, particularly in the range of 600 cm^-1^-2000 cm^-1^. However, there are three peaks at between 1500 cm^-1^ and 1700 cm^-1^ which are merely related to the PA layer. These PA characteristic peaks were highlighted in a separate window in panel (b). The peak at 1667 cm^-1^ is attributed to C=O stretching vibration (amide I bands), the peak at 1611 cm^-1^ belongs to aromatic amide ring breathing, and the peak at 1541 cm^-1^ is associated (mainly) with N-H bending as well as the C-N stretching vibration (amide II bands) of the -CO-NH-group ^57^. The broad peak at 3300 cm^-1^ is due to stretching vibration of the N-H groups in the PA layer ^58,59^.


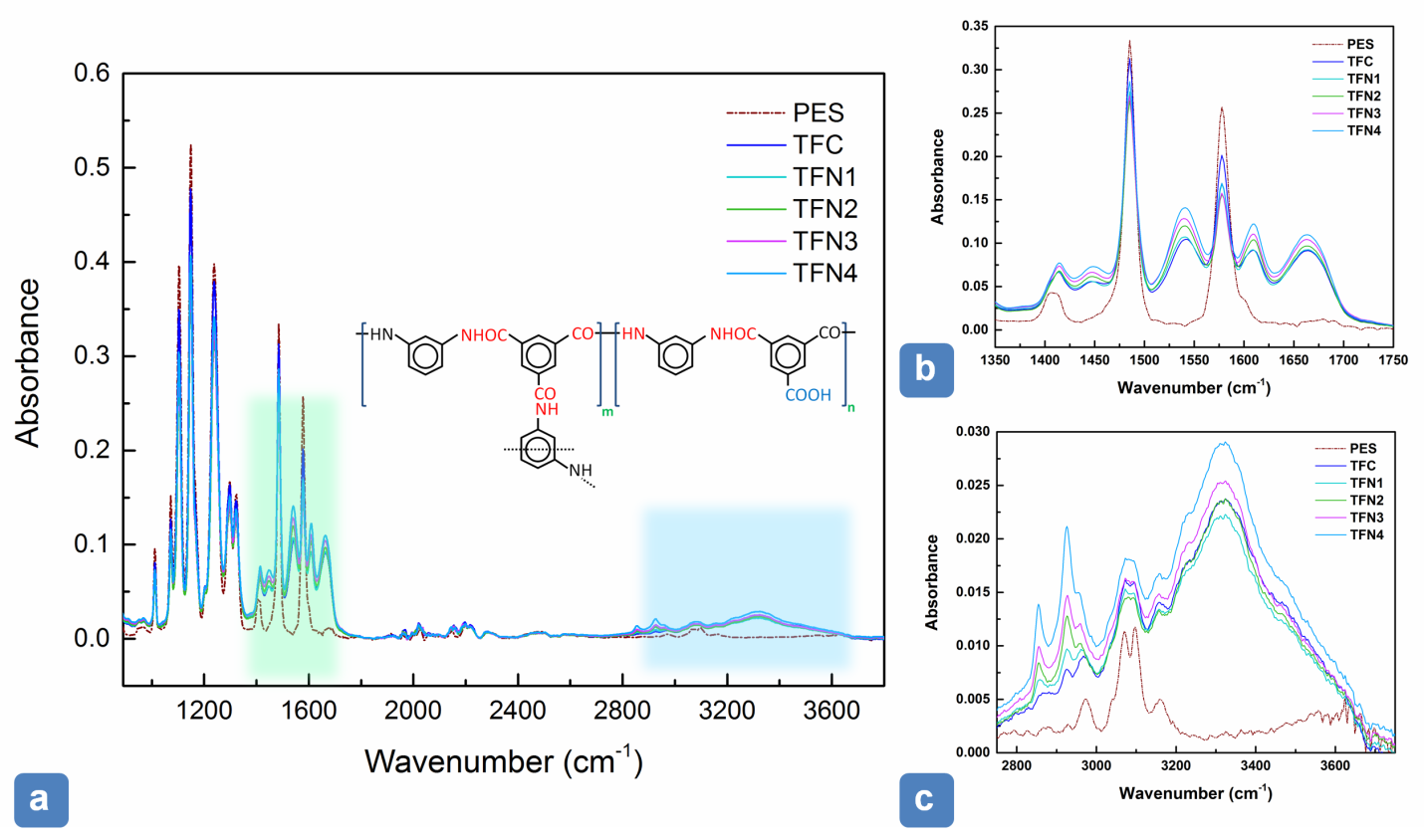


Figure S1: (a) The ATR-FTIR spectroscopy of the synthesized TFC and TFN membranes; (b) PA characteristic peaks emerge at 1541 cm^-1^, 1611 cm^-1^, and 1667 cm^-1^ attributing to N-H bending and C-N stretching vibration of amide II (–CONH-) group, aromatic ring breathing and C=O stretching vibration of amide I bands, respectively; (c) The broad peak at 3300 cm^-1^ is formed to stretching vibration of the N-H groups in the PA layer.

EDX spectroscopy was utilized to analyze the chemical composition of the TFN membranes, and the results are presented in Figure S2 for TFN2 and TFN4 membranes. In addition, the elemental composition was evaluated at two different spots: TiO_2_ rich (point A, white region in the FESEM image with BSC detector); and lean (Point B, dark region in the FESEM image with BSC detector) spots at the surface of TFN2 and TFN4 membranes. The EDX spectra illustrate a distinct peak for titanium at the membrane surface, particularly at point A where the blend of PA and TiO_2_ NPs formed large clusters at the surface. A comparison between the composition results of the TFN2 and TFN4 membranes shows that the weight percentage of the TiO_2_ NPs at the PA surface increased with rise in the concentration of the TiO_2_ NPs in the TMC solution. This result implies the effective integration of the TiO_2_ NPs during the IP reaction.


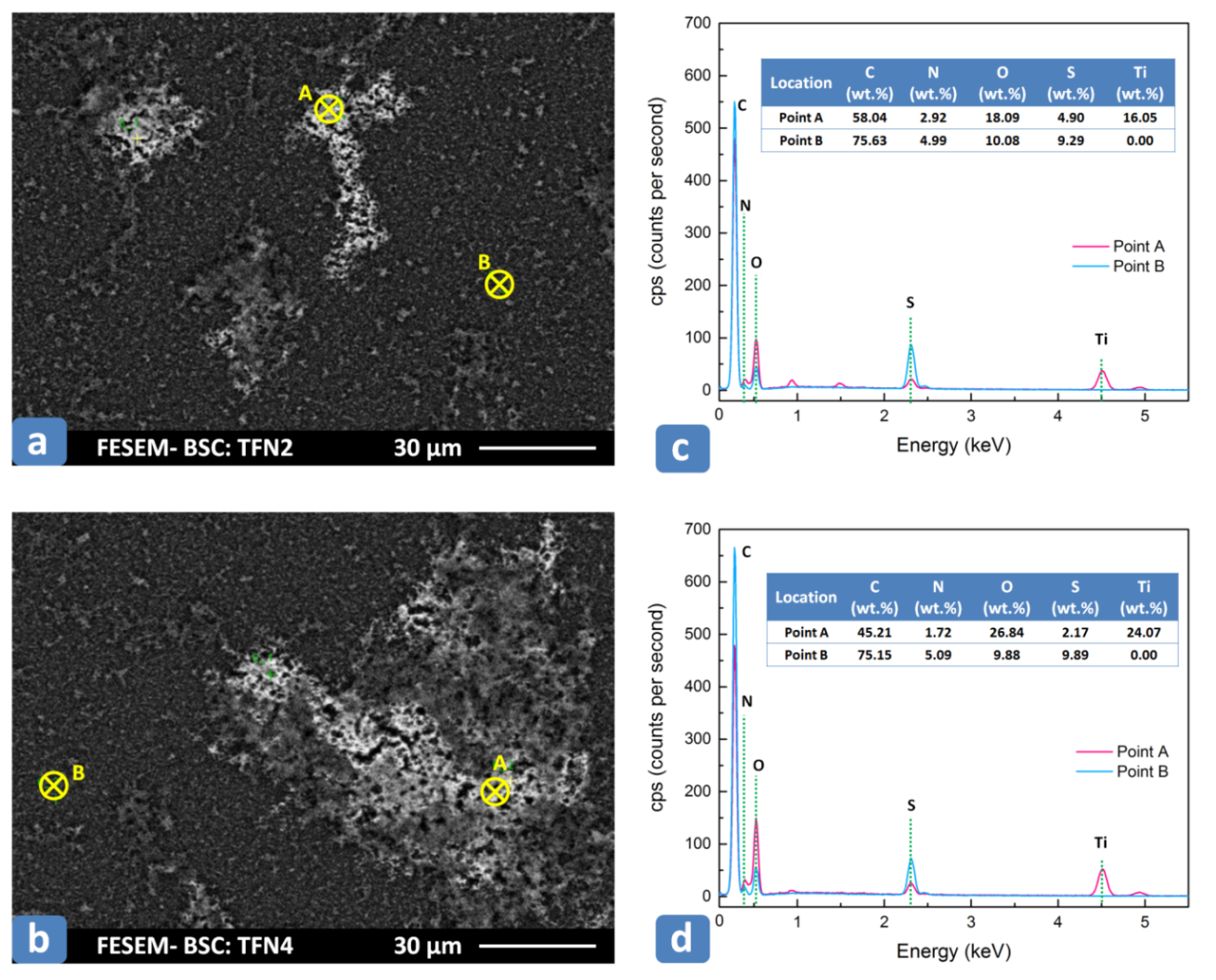


Figure S2: (a) and (b) FESEM image with BSE detector of TFN2 and TFN4 membranes, respectively; (c) and (d) EDX spectra at TiO_2_ rich (point A) and lean (point B) spots at the surface of TFN2 and TFN4 membranes, respectively.

The thermomechanical stability of the bse TFC and TFN4 membranes was also tested by thermogravimetric analysis (TGA) and the results are presented in Figure S3. Based on this figure, the onset of intense degradation temperature of the composite membranes has slightly increased from 530 °C for the base TFC to 550 °C for the TFN4 memrbanes. The improved thermal stability is attributed to the decreased polymer chain mobility due to the presence of nano-sized and homogeneously distributed TiO_2_ NPs.


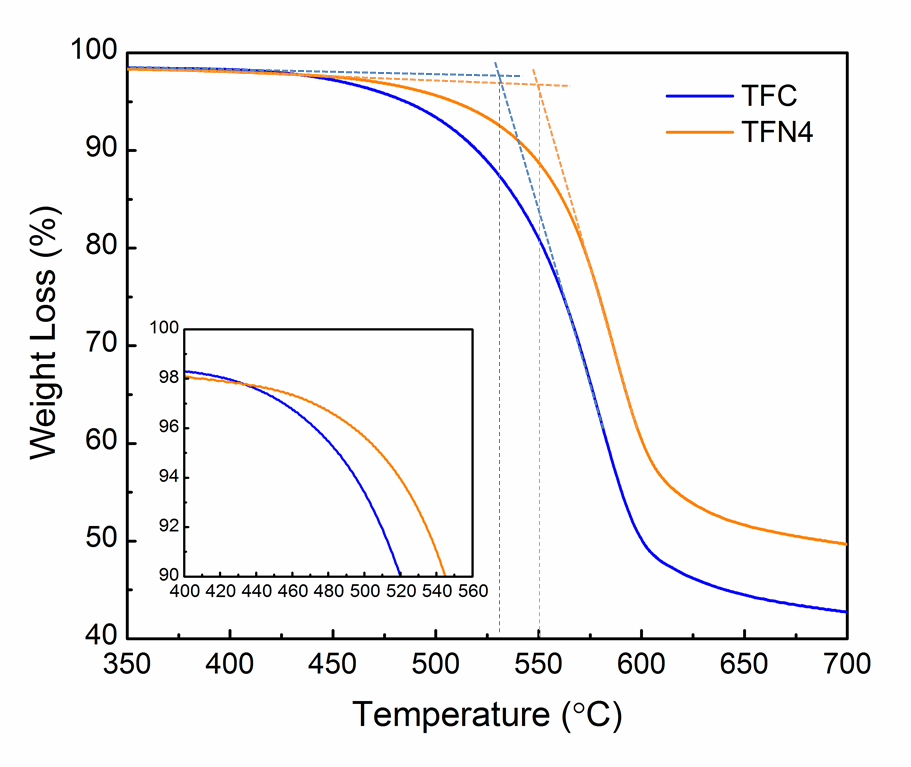


Figure S3: Thermogravimetric analysis (TGA) of base TFC and TFN4 membranes. The dashed lines show the onset of intense thermal degradation of the polymers.

1. ^^ Corresponding Author: [sadrzade@ualberta.ca](mailto:sadrzade@ualberta.ca) [↑](#footnote-ref-2)
